# A Heterozygous Deletion Mutation in the Cardiac Sodium Channel Gene SCN5A with Loss- and Gain-of-Function Characteristics Manifests as Isolated Conduction Disease, without Signs of Brugada or Long QT Syndrome

**DOI:** 10.1371/journal.pone.0067963

**Published:** 2013-06-28

**Authors:** Sven Zumhagen, Marieke W. Veldkamp, Birgit Stallmeyer, Antonius Baartscheer, Lars Eckardt, Matthias Paul, Carol Ann Remme, Zahurul A. Bhuiyan, Connie R. Bezzina, Eric Schulze-Bahr

**Affiliations:** 1 Institute for Genetics of Heart Diseases (IfGH), Department of Cardiovascular Medicine, University Hospital Münster, Münster, Germany; 2 Clinical and Experimental Cardiology, Heart Center, Academic Medical Center, Amsterdam, The Netherlands; 3 Division of Electrophysiology, Department of Cardiovascular Medicine, University Hospital Münster, Münster, Germany; 4 Division of Cardiology, Department of Cardiovascular Medicine, University Hospital Münster, Münster, Germany; 5 Laboratoire de Génétique Moléculaire, Service de Génétique Médicale, Centre Hospitalier Universitaire Vaudois (CHUV), Lausanne, Switzerland; 6 Interdisciplinary Centre for Clinical Research (IZKF), University Hospital Münster, Münster, Germany; Cedars-Sinai Medical Center, United States of America

## Abstract

**Background:**

The *SCN5A* gene encodes for the α-subunit of the cardiac sodium channel Na_V_1.5, which is responsible for the rapid upstroke of the cardiac action potential. Mutations in this gene may lead to multiple life-threatening disorders of cardiac rhythm or are linked to structural cardiac defects. Here, we characterized a large family with a mutation in *SCN5A* presenting with an atrioventricular conduction disease and absence of Brugada syndrome.

**Method and Results:**

In a large family with a high incidence of sudden cardiac deaths, a heterozygous *SCN5A* mutation (p.1493delK) with an autosomal dominant inheritance has been identified. Mutation carriers were devoid of any cardiac structural changes. Typical ECG findings were an increased P-wave duration, an AV-block I° and a prolonged QRS duration with an intraventricular conduction delay and no signs for Brugada syndrome. HEK293 cells transfected with 1493delK showed strongly (5-fold) reduced Na^+^ currents with altered inactivation kinetics compared to wild-type channels. Immunocytochemical staining demonstrated strongly decreased expression of *SCN5A* 1493delK in the sarcolemma consistent with an intracellular trafficking defect and thereby a loss-of-function. In addition, *SCN5A* 1493delK channels that reached cell membrane showed gain-of-function aspects (slowing of the fast inactivation, reduction in the relative fraction of channels that fast inactivate, hastening of the recovery from inactivation).

**Conclusion:**

In a large family, congregation of a heterozygous *SCN5A* gene mutation (p.1493delK) predisposes for conduction slowing without evidence for Brugada syndrome due to a predominantly trafficking defect that reduces Na^+^ current and depolarization force.

## Introduction

The *SCN5A* gene is located on the short arm of chromosome 3 (3p21), contains 28 exons and encodes for the α-subunit of the cardiac sodium channel (Na_V_1.5), that has a weight of ∼220 kDa and consists of 2,016 amino acids [1], [2], [3]. The Na_V_1.5 α-subunit is the pore-forming component of the Na_V_1.5 channel and contains four homologous transmembrane domains (DI to DIV) joined by three linkers. Each of the domains consists of six transmembrane segments (S1 to S6) linked by intra- or extracellular loops [4], [5], [6]. S4 is positively charged and is involved in voltage-dependent activation of the channel, while inactivation is mediated mainly by the DIII–DIV linker [4]. Na_V_1.5 is responsible for the upstroke (phase 0) of the action potential of cardiac cells. Opening of the channel leads to a rapid influx of positive charged Na^+^ ions (I_Na_), which will depolarize the membrane potential within tenths of a millisecond [7]. I_Na_ plays a central role in the initiation, propagation, as well as cardiac excitation of the cardiac impulse [8]. Overall, the cardiac sodium channel is a multiprotein complex in which auxiliary proteins interact with α-subunit (Na_V_1.5) encompassing enzymes, regulatory proteins and adaptor proteins that modulate gating properties, cellular localization, regulate intracellular transport, targeting and degradation of Na_V_1.5 [5], [7].

Na_V_1.5 channels are located in the sarcolemma of atrial and ventricular myocytes, the Purkinje fibers and to a lesser extent in the sinoatrial and atrioventricular node [6]. Mutations in *SCN5A* lead to various arrhythmogenic diseases, e.g. long QT syndrome (LQTS; subform LQT-3), Brugada syndrome (BrS; BrS-1), cardiac conduction disease (CCD, also known as Lev-Lenègre syndrome), but also idiopathic atrial fibrillation, sinus node dysfunction, atrial standstill, and even dilated cardiomyopathy (DCM) [9], [10], [11], [12], [13], [14], [15], [16], [17]. This emphasizes the phenotypical heterogeneity of *SCN5A* mutations, and overlapping clinical and in-vitro phenotypes [18]. The basic cause is a change in Na_V_1.5 expression and biophysical properties leading to a loss-of-function or gain-of-function by various mechanisms. Interestingly, overlap syndromes have also been reported, primarily for *SCN5A* mutations leading to BrS [16], [19], [20], [21].

Here we investigated functional consequences of the 1493delK *SCN5A* mutation, which was identified in a clinically characterised large family with a high incidence of sudden cardiac deaths (SCD). The positively charged lysine residue is located in the DIII–DIV linker, close to the inactivation particle, and is expected to modulate sodium channel fast inactivation.

## Methods

### Ethics Statement

This study was approved by the Ethics Committee of the University Hospital Münster (Münster, Germany) and conforms to the principles outlined in the Declaration of Helsinki [22]. All probands and their relatives who participated in the study gave written informed consent before genetic and clinical investigations.

### Study Population

Detailed clinical data, including cardiac symptoms, device implantation, standard 12-lead ECGs, and cardiac imaging (transthoracic echocardiography, magnetic resonance imaging with gadolinium contrast, or ventriculography) were obtained. ECG analysis was performed mainly upon conventional 12-lead ECG recordings with standard lead positions (paper speed 25 or 50 mm/s). Recordings were digitalized by scanning in a high-resolution format and were imported into a graphic program (DatInf® Measure, Germany) for accurate measurements. Duration of the PQ, QRS and QT intervals as well as RR intervals for calculating the heart rate were measured in three consecutive beats and provided as mean values. ECG and arrhythmia analyses were independently performed by two cardiologists.

### Genetic Analysis

Genetic analysis was performed as previously described [23]. In brief, after isolation of genomic DNA, all 28 exons and adjacent intronic sequences of the cardiac sodium channel gene (*SCN5A*) were analysed by sequencing (ABI3500, Applied Biosystems, Germany) after directed and selective PCR amplification using standard procedures. Obtained nucleotide sequences were compared with published wild-type sequences (nucleotide sequence: GenBank, NM_000335; protein sequence: Swiss-Prot, entry Q14524). Identified sequence variations were cross-checked for their occurrence in the SNP database of NCBI (http://www.ncbi.nlm.nih.gov/projects/SNP), the Exome Variant Server (http://evs.gs.washington.edu/EVS/), disease specific mutation databases (http://www.fsm.it/cardmoc/; http://www.genomed.org/lovd/lqts/home.php?select_db=SCN5A) and PUBMED entries. DNA from 380 healthy, unrelated individuals was used to demonstrate absence in a control sample.

### Heterologous Expression of Cardiac Na_V_1.5 Channels

*SCN5A* cDNA was cloned into the pCGI vector for bicistronic expression of Na_V_1.5 and green fluorescent protein, as described previously [24]. Human embryonic kidney (HEK293) cells were transiently transfected with 0.5 µg of wild-type (WT) or 1493delK mutant Na^+^ channel α-subunit cDNA, together with 0.5 µg hβ_1_-subunit cDNA (kindly provided by J.R. Balser, Vanderbilt University, Nashville, Tenn) using lipofectamine (Gibco BRL, Life Technologies). Transfected HEK293 cells were cultured for 1 or 2 days, as described previously [25]. Only cells exhibiting green fluorescence were selected for electrophysiological experiments.

### Electrophysiology and Data Analysis

Sodium currents were measured at room temperature (23°C) in the whole-cell configuration of the patch-clamp technique using an Axopatch 200B amplifier (Axon Instruments) and the following solutions (in mmol/L): bath (external) solution: NaCl 140, KCl 4.7, CaCl_2_ 1.8, MgCl_2_ 2.0, NaHCO_3_ 4.3, Na_2_HPO_4_ 1.4, glucose 11, and HEPES 16.8, pH adjusted to 7.4 (NaOH); and pipette (internal) solution: CsF 100, CsCl 40, EGTA 10, NaCl 10, MgCl_2_ 1.2, and HEPES 10, pH adjusted to 7.3 (NaOH). Patch electrodes were pulled from borosilicate glass and had a tip resistance of 2 MΩ when filled with pipette solution. Series resistance was compensated for ≥80%. For current density measurements, all cells were included. For measurements of gating properties, adequate voltage control was ascertained by the presence of a graded slope of the (in)activation curve (Boltzmann slope factors: >4.5 and >4.0 for activation and inactivation, respectively). Experiments were further excluded from analysis when peak sodium current amplitudes at −20 mV were <0.6 nA or >10 nA. Currents were filtered at 5 kHz and digitized at 20 kHz. Voltage control, data acquisition, and analysis were performed with a custom-made software (kindly provided by A.C.G. van Ginneken and J.G. Zegers). Voltage-dependence of activation and steady-state inactivation, recovery from inactivation, and development of slow inactivation were determined using voltage-clamp protocols depicted in the relevant figures. In all protocols, a holding potential of −120 mV and a cycle time of 5 s was used. Voltage-dependence of activation and inactivation curves were fitted with a Boltzmann function (*y* = [1+ exp{(*V*−*V*_1/2_)/*k*}]^−1^), where *V*_1/2_ is the half-maximal voltage of (in)activation, and *k* is the slope factor. Recovery from inactivation was assessed with a double-pulse protocol. Data were normalized to the current elicited by the first pulse (*P*_1_) and fitted with a bi-exponential function (*y* = *y*_0_+ *A*_f_{1−exp[−*t*/*τ*_f_]}+*A*_s_{1−exp[−*t*/*τ*_s_]}), where *A*_f_ and *A*_s_ represent the amplitudes of the fast and the slow components of recovery from inactivation, respectively, and *τ*_f_ and *τ*_s_ are their respective recovery time constants. *I*_Na_ decay was fitted with a bi-exponential function (*y* = *y*_0_+ *A*_f_exp[−*t*/*τ*_f_]+*A*_s_exp[−*t*/*τ*_s_]), where *A*_f_ and *A*_s_ are the amplitudes of the fast and the slow inactivating components, respectively, and *τ*_f_ and *τ*_s_ are their respective inactivation time constants. Development of slow inactivation was fitted with a single exponential function *y* = *A*+*A*_0_exp^−*t*/*τ*^, where *τ* is the time constant for the development of slow inactivation, and *A* is the fraction of channels that enter the slow-inactivated state after a 1000 ms depolarization step.

### Immunocytochemistry

Transfected HEK293 cells were grown on glass coverslips coated with 0.1% laminin. Before fixation, coverslips with cultured cells attached were rinsed in serum-free medium, fixed in 4% paraformaldehyde (PFA) for 15 min at room temperature (RT), and washed wit phosphate buffered saline (PBS). Next, cells were incubated in 0.1% Triton X-100 in PBS for 30 min at RT. Cells were then washed twice with PBS and blocked with 2% BSA (in PBS) for 30 min at RT. Primary antibodies anti-Na_V_1.5 (1∶300) and anti-calnexin (1∶500) were added to each coverslip and incubated overnight at RT. After washing 3 times with PBS and blocking in 2% BSA for 30 min, cells were incubated with the secondary antibody for 90 min (both at RT). After 3 additional washings with PBS, the coverslips were embedded over glass slides using glycerol. Confocal imaging was performed using the Leica SPE confocal laser scanning microscope (scanning enlargement 40X with additional 3X digital zoom. For dual labeling experiments, dual color red and green images were recorded.

### Antibodies

Anti-Na_V_1.5 (Alomone Laboratories, ASC-005) is a rabbit polyclonal antibody raised against a peptide corresponding to residues 492–511 of rH1. The mouse monoclonal anti-calnexin antibody (Chemicon International, MAB3126) was used for labeling of endoplasmic reticulum (ER). Alexa conjugated goat anti- mouse and anti-rabbit antibodies (Molecular Probes, Invitrogen) were used as secondary antibodies.

### Statistical Analysis

Results are expressed as mean ±SEM. Comparisons were made using unpaired Student's *t-*test or Mann–Whitney test when data were not normally distributed, and two-way ANOVA with repetitive measurements followed by a Holm–Sidak test for *post-hoc* analysis, where appropriate. *p*<0.05 indicates statistical significance.

## Results

### Clinical Data and Molecular Genetics

**Figure 1:**
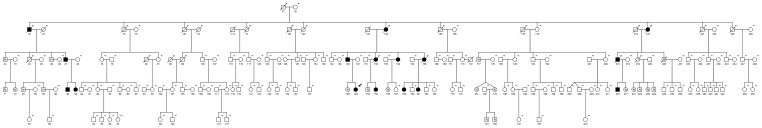
Pedigree of the family 10021. Men are denoted by squares and women by circles. Solid symbols indicate mutation carriers, symbols with an “N” means wild type and “?” possible affected, crossed symbols denotes patients, who are already dead. A “-“ at the symbols indicates that no DNA is available, the propositus is marked with an arrow.

**Figure 2:**
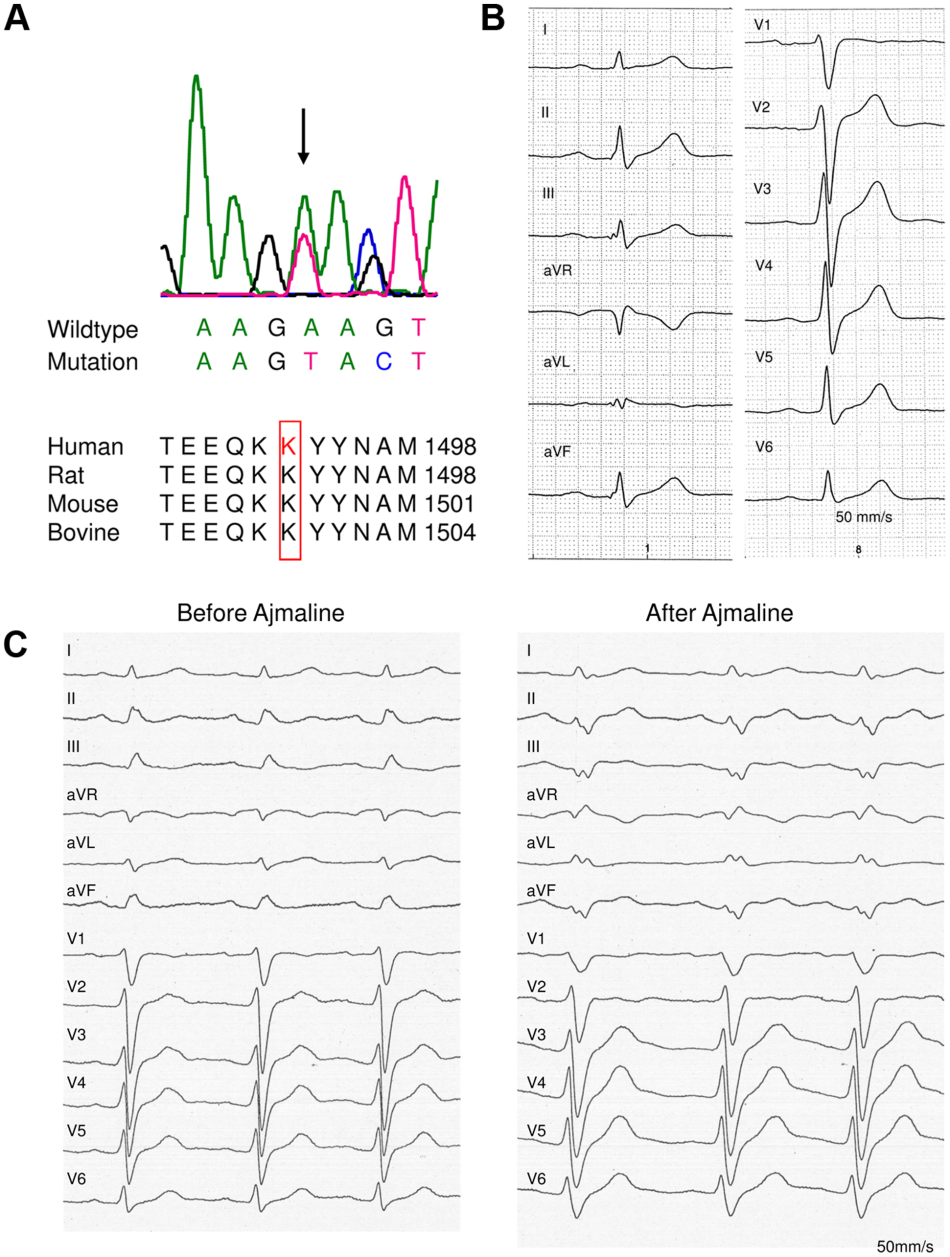
Clinical and genetic characterization. (A) Electropherogram of *SCN5A* mutation c.4477–4479delAAG and multiple sequence alignment of amino acids of human *SCN5A* protein regions bearing the identified in-frame deletion mutation of lysine (p.1492delK) with corresponding *SCN5A* amino acid sequences of different species. (B) Electrocardiogram of patient 10021_49 shows an atrioventricular block first-degree, an increased P-wave duration and an intraventricular conduction delay (P interval 145 ms, PQ interval 208 ms, QRS interval 146 ms). (C) Ajmaline challenge of patient 10021_149, overall 54 mg ajmaline (1mg/kg) was administered within 5 minutes. No Brugada type I ECG could be unmasked, but cardiac conduction delay aggravated.

In a large family (family ID: 10021) with 182 members (Figure 1) and a high incidence of sudden cardiac death (n = 8), a genetic and clinical screening was performed in 30 patients. In 10 cases of this patient group (4 males, 6 females; mean age 56.2±16.5 years), a novel heterozygous *SCN5A* mutation (c.4477–4479delAAG; p.1493delK) with an autosomal dominant inheritance was identified. This mutation was previously assigned as p.1479delK [23] and later corrected into p.1493delK [26]. From 6 additional affected family members DNA for genetic analysis was not available, but they were obligate mutation carriers. At the protein level, the mutation is predicted to cause an in-frame deletion of a lysine at position 1493 of the Na_V_1.5 open reading frame due to a loss of the nucleotide triplet AAG at position 4477 to 4479 (Figure 2A). This amino acid residue is located in the linker between the domains DIII S6 and DIV S1 that is known to be responsible for the inactivation of the channel [27]. In addition, this mutation was not detected in 380 European control samples and neither in >12,000 alleles screened in the setting of the NHLBI Go Exome Sequencing Project (Exome Variant Server, Seattle, WA; URL: http://evs.gs.washington.edu/EVS/[accessed February 2013]). The lysine residue at position 1493 was shown to be orthologous highly conserved. Brief clinical information of the identified mutation carriers as well as the obligate mutation carriers is presented in Table 1. Overall, the 10 mutation carriers showed no signs for gross structural heart disease by transthoracic echocardiography, magnetic resonance imaging or left/right ventriculography [28]. Ajmaline challenge performed in six patients proven to carry the *SCN5A* mutation, did not elicit a Brugada type I-ECG that is diagnostic for BrS. However, all mutation carriers showed signs of CCD in rest ECG (Figure 2B). An increased P-wave duration (122.1±11.1 ms in lead II, 119.7±15.8 ms in lead V2 and 122.1±19.8 in lead V5; normal 50–100 ms) was identified in all patients (Table 2) and an intraventricular conduction delay in 9 out of 10 patients characterized by prolonged QRS intervals (112.9±24.1 ms in lead II, 130.3±22.5 ms in lead V2 and 114.7±21.4 ms in V5) and with no signs for right or left fascicular block (except for patient (10021_47). Furthermore, 5 out of 10 mutation carriers presented with an AV-block I° (194.4±19.9 ms in lead II, 188.9±24.5 ms in lead V2, 190.8±27.7 ms in lead V5).

**Table 1:** Clinical and Genetic Characteristics of *SCN5A* 1493delK Carriers.

Patient ID	Gender	Age (y)	SCN5A Mutation	Clinical Presentation	Cardiac Imaging	Device	ECG	Ajmaline Challenge
10021_47	m	60	+	asymptomatic	normal^#,§,$^	ICD	increased PWD, AVB I°, CCD, RFB, LAFB	exclusion of BrS
10021_49	m	30	+	asymptomatic	normal^#^	–	increased PWD, AVB I°, CCD	n.p.
10021_50	f	35	+	asymptomatic	normal^#^	–	increased PWD, CCD	exclusion of BrS
10021_146	m	51	+	asymptomatic	normal^#^, mild RV-hypokinetic^§^,1-vessel CAD^$^	ICD	increased PWD, AVB I°, CCD, ST-elevation in V1/V2	n.p.
*10021_149*	*f*	*18*	*+*	*asymptomatic*	*normal^#,§,$^*	*ICD*	*increased PWD, AVB I°, CCD*	*exclusion of BrS*
10021_153	f	28	+	asymptomatic	normal^#^	–	increased PWD	n.p.
10021_154	f	57	+	asymptomatic	normal^#^	ICD	increased PWD, CCD	n.p.
10021_160	f	18	+	asymptomatic	normal^#^	–	increased PWD, CCD	exclusion of BrS
10021_161	f	20	+	asymptomatic	normal^#^	–	increased PWD, CCD	exclusion of BrS
10021_218	m	20	+	asymptomatic	normal^§,$^	ICD	increased PWD, AVB I°, CCD	exclusion of BrS
10021_33	m	73	(+)	non-cardiac death	n.a.	–	n.a.	n.p.
10021_142	f	79	(+)	natural death	n.a.	–	n.a.	n.p.
10021_150	f	49	(+)	SCD	n.a.	PM	n.a., sinus bradycardia reported	n.p.
10021_158	f	47	(+)	SCD	n.a.	–	increased PWD, CCD	n.p.
10021_213	f	68	(+)	non-cardiac death	n.a.	–	n.a.	n.p.
10021_215	m	44	(+)	SCD	n.a.	–	n.a.	n.p.

ECG, electrocardiogram; ICD, implantable cardioverter defibrillator; PM, Pacemaker; m, male; f, female; y, years; +, mutation carrier or ICD implanted; (+),obligate mutation carrier; SCD, sudden cardiac death; RV, right ventricle; CAD, coronary artery disease; #, by transthoracic echocardiographic; §, by magnetic resonance imaging; $, by ventriculography; n.p., not performed; n.a., not avalible; PWD, P-wave duration; AVB I°, atrioventricular block first-degree; CCD, cardiac conduction delay; RFB, right fascicular block; LAFB, left anterior fascicular block. Propositus (10021_149) printed in italics.

**Table 2:** Electrocardiogram Parameters of Mutation or Obligate Mutation Carriers.

Patient ID	Gender	Lead II					Lead V2					Lead V5				
		HR [bpm]	P [ms]	PQ [ms]	QRS [ms]	QTc [ms]	HR [bpm]	P [ms]	PQ [ms]	QRS [ms]	QTc [ms]	HR [bpm]	P [ms]	PQ [ms]	QRS [ms]	QTc [ms]
10021_47	m	54	120	222	166	416	55	134	232	148	395	55	153	244	158	410
10021_49	m	67	142	208	123	385	72	145	206	146	390	72	103	178	115	372
10021_50	f	87	120	193	99	458	88	114	168	106	435	87	122	184	101	436
10021_146	m	81	131	219	91	423	80	115	214	115	426	80	137	215	109	431
10021_149	f	76	131	203	124	434	74	132	193	151	444	71	143	222	129	427
10021_153	f	73	115	174	92	394	73	97	160	96	392	73	101	157	93	393
10021_154	f	not measureable					not measureable					not measureable				
10021_158	f	64	101	178	109	396	64	101	192	127	382	64	93	180	112	392
10021_160	f	68	121	168	83	355	68	122	157	107	378	68	118	160	82	358
10021_161	f	69	114	174	120	411	62	106	172	155	388	62	116	181	118	381
10021_218	m	50	127	205	124	384	51	132	194	151	382	51	137	188	130	373
Mean values ±SD		69.0±11.2	122.1±11.1	194.4±19.9	112.9±24.1	405.5±29.3	68.6±11.1	119.7±15.8	188.9±24.5	130.3±22.5	401.2±24.4	68.4±11.1	122.1±19.8	190.8±27.7	114.7±21.4	397.2±27.5

HR, heart rate; P, P interval; PQ, PQ interval; QRS, QRS interval; QTc, corrected QT interval; m, male; f, female; SD, standard deviation.

In the group with the obligate mutation carriers (2 males, 4 females; mean age 60.0±15.1 years) SCD - defined as an unexpected death due to cardiac cause, without known cardiac diseases and under the age of 50 years - occurred in 3 cases. In one of these cases (10021_158, proband) an ECG was available, in which a wide P-wave (101 ms in lead II and lead V2) and an intraventricular conduction delay (QRS interval 109 ms in lead II, 127 ms in lead V2 and 112 ms in lead V5) without classical signs for right or left fascicular block was documented. However, this ECG was recorded nearly 18 years before SCD occurred and cardiac conduction slowing may have further worsened in the course of time. In summary, mutation carriers showed cardiac conduction delay but no signs for Brugada syndrome (ajmaline challenge inconspicuous, Figure 2C) or repolarisation disturbance (normal QTc intervals).

### Electrophysiological Characterization of 1493delK Mutant Sodium Channels

**Figure 3:**
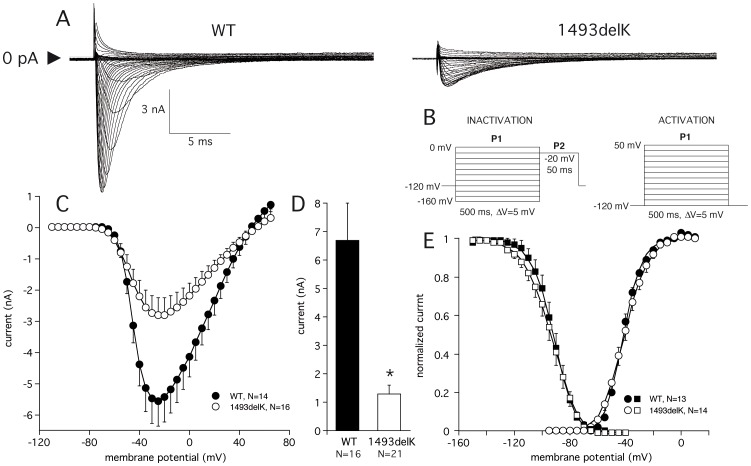
1493delK mutant and wild-type (WT) human cardiac sodium channel current expressed in HEK293 cells. (A) Whole-cell sodium current traces in response to increasing step depolarizations in WT (left) and 1493delK (right). (B) Voltage protocols for activation and steady-state inactivation. (C) Averaged sodium current– voltage relation for WT and 1493delK sodium channels. (D) Bar histogram showing averaged WT and 1493delK sodium peak currents at −20 mV. (E) Average voltage-dependence of activation and steady-state inactivation for wild-type (WT) and 1493delK sodium channels. For the activation curve, normalized peak conductance was plotted as a function of the membrane potential. For the inactivation curve, peak sodium currents were normalized to maximum values in each cell and plotted as a function of the voltage of the conditioning step.

**Figure 4:**
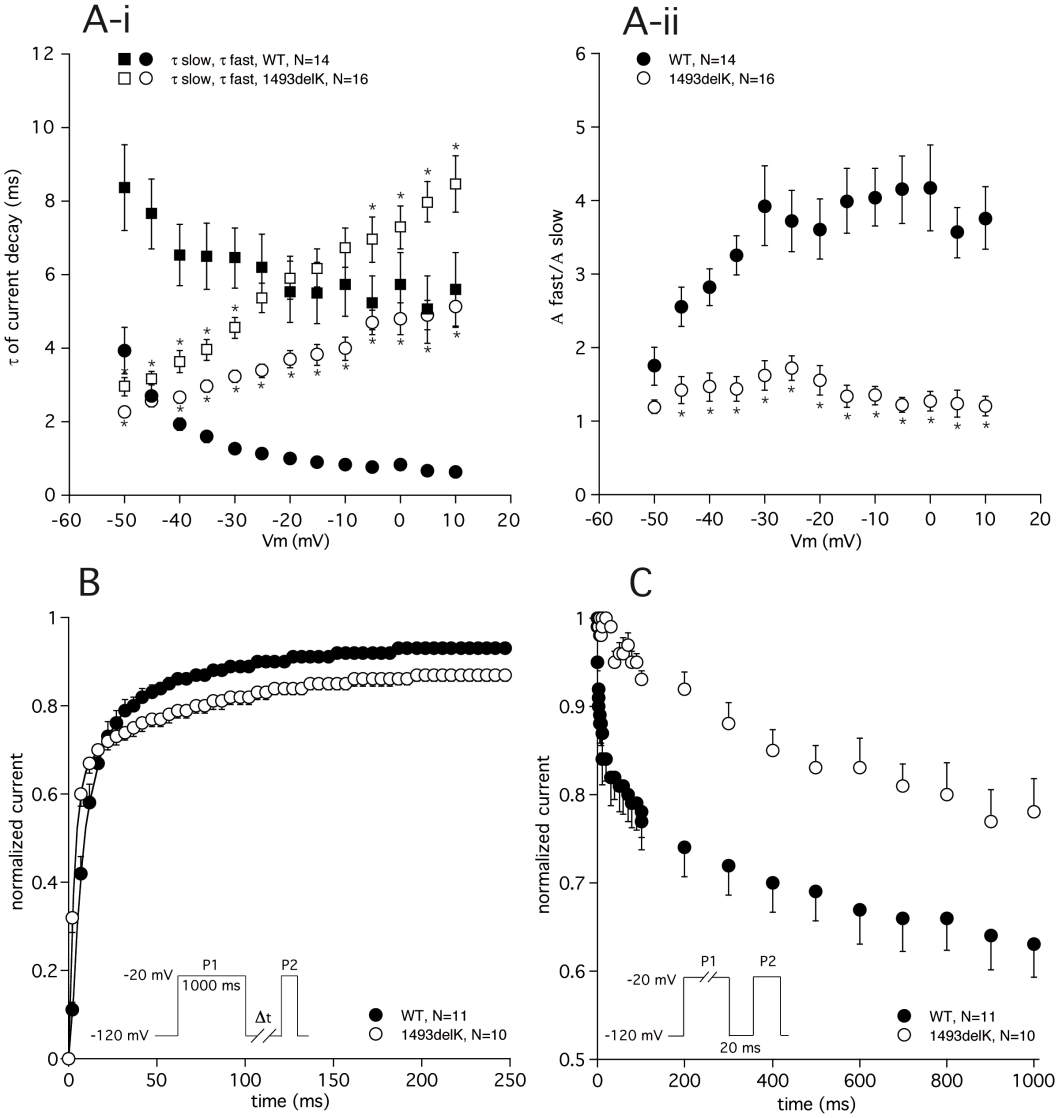
Inactivation kinetics of 1493delK mutant and wild-type (WT) human cardiac sodium channels. (A) Time course of current decay. (A-i) Fast and slow time constants of current decay for WT and 1493delK sodium channels are plotted as a function of membrane potential. Asterisks indicate statistical significance (p<0.05). (A-ii) Ratio of the amplitudes of fast and slow inactivation time constants plotted as a function of voltage for WT and 1493delK sodium channels. (B) Time course of recovery from inactivation for WT and 1493delK sodium channels. Peak sodium currents elicited by P2 were normalized (P2/P1) and plotted as a function of the recovery interval. Inset: 2-pulse protocol. (C) Development of slow inactivation for WT and 1493delK sodium channels. Peak sodium currents elicited by P2 were normalized (P2/P1) and plotted as a function of the duration of the conditioning step (P1). Inset: 2-pulse protocol.

To assess the functional consequences of the 1493delK mutation, sodium current (I_Na_) biophysical properties were determined in HEK293 cells expressing wild-type (WT) and 1493delK mutant sodium channels. Results are summarized in Table 3. Comparison of WT and 1493delK mutant peak I_Na_ recorded during step depolarization (Figure 3A), and the corresponding average current–voltage (I–V) relationships (Figure 3C), clearly reveal smaller current magnitudes for 1493delK mutant channels. The average amplitude of the maximum peak I_Na_ was 5.6±0.8 nA (n = 14) for WT and 2.8±0.5 nA (n = 16) for 1493delK mutant channels (Figure 3C, Table 3). Inclusion of all experiments, i.e. also experiments with I_Na_ <0.6 nA and >10 nA (see Methods: data analysis) amounted to a five-fold reduction in I_Na_ at −20 mV for 1493delK mutant I_Na_ as compared to WT (Figure 3D, WT: 6.7±1.3 nA vs. 1493delK: 1.3±0.3 nA). Investigation of the voltage-dependence of activation and steady-state inactivation did not reveal significant differences between WT and 1493delK mutant sodium channels. The half-maximal voltage V_1/2_ of activation and inactivation was similar in both groups, with a slight increase of the slope factor k (Figure 4E, Table 3).

**Table 3:** Electrophysiological characteristics of WT and 1493delK mutant sodium channels in HEK293 cells.

	WT	1493delK
**I_Na_ amplitude (nA):**	(n = 16)	(n = 21)
At −20 mV	–6.7±1.3	–1.3±0.3*
**Activation:**	(n = 14)	(n = 16)
V_1/2_ (mV)	–40.7±1.1	–39.5±1.7
k (mV)	6.2±0.3	7.5±0.4*
**Steady-state inactivation:**	(n = 13)	(n = 14)
V_1/2_ (mV)	–92.5±2.4	–94.0±2.3
k (mV)	–6.3±0.4	–7.5±0.3*
**Recovery from inactivation:**	(n = 10)	(n = 10)
* τ*_f_ (ms)	8.9±1.2	3.8±0.4*
* τ*_s_ (ms)	93.2±24.9	82.8±6.4
**Development of slow inactivation:**	(n = 9)	(n = 11)
* τ* (ms)	182.3±22.3	825.8±197.5*
A (ms)	0.37±0.04	0.22±0.4*

V_1/2_, voltage of half-maximal (in)activation; k, slope factor of voltage- dependence of (in)activation; A, fraction of channels that enter the slow inactivated state at t = 1 s; *τ*, time constant for development of slow inactivation; *τ*_f_, fast time constant of recovery from inactivation; *τ*_s_, slow time constant of recovery from inactivation.

* p<0.05 vs WT (Student’s t-test).

Since the DIII–DIV linker has been shown [29] to be responsible for channel fast inactivation, the deletion of a charged residue from this region is likely to affect inactivation kinetics. In Figures 4A and 3B, the time-dependent properties of the fast inactivation process are summarized. Close inspection of the 1493delK current tracings in Figure 3B suggests a slowing of inactivation, which was further supported by fitting the time course of current decay at various voltages with a bi-exponential function. The 1493delK mutant sodium channels displayed a remarkable and atypical voltage dependence of the inactivation time constants (Figure 4A-i). While fast (*τ*_f_) and slow (*τ*_s_) time constants of current inactivation of WT sodium channels are typically decreased on incremental depolarization, inactivation time constants of 1493delK mutant sodium channels were increased. Although *τ*_f_ in 1493delK is smaller at −50 mV, there is a cross-over at −40 mV from where on *τ*_f_ exceeds that of WT sodium channels, thus decreasing inactivation rate. The *τ*_s_ of inactivation in 1493delK was similarly affected, but with a less negative cross-over voltage of −20 mV. In addition to slowing the inactivation rate, the 1493delK mutant also affected the relative contribution of the fast inactivation component to the overall time course of current decay. Figure 4A-ii shows the ratio - expressed as a quotient - of the amplitudes of the fast and the slow inactivating components (*A*_f_ and *A*_s_) as a function of voltage. In case of WT sodium channels, time course of current decay is largely determined by the fast inactivating component, constituting ∼75% of total I_Na_ (Figure 4A-ii). The 1493delK mutation strongly reduced the contribution of the fast component to the inactivation time course, giving rise to a decrease in the ratio of *A*_f_/*A*_s_ at 0 mV from 4.2±0.6 (WT, n = 14) to 1.3±0.1 (1493delK, n = 16). In summary, the overall effect of the 1493delK mutation on inactivation parameters is a profound slowing of the inactivation rate.

Additionally, we determined the rate of recovery from inactivation at −120 mV using a two-pulse protocol. The recovery time course of inactivation was significantly accelerated for 1493delK sodium channels as compared to WT sodium channels (Figure 4B, Table 3). This was exclusively due to a reduction in *τ*_f_ value for 1493delK sodium channels (1493delK: *τ*_f_  = 3.8±0.4 ms vs. WT: *τ*_f_  = 8.9±1.2 ms); values of *τ*_s_ were comparable (1493delK: *τ*_s_  = 82.5±65.3 ms vs. WT: *τ*_s_  = 93.2±24.9 ms).

On sustained depolarization, cardiac sodium channels tend to enter a ‘slow’ or ‘intermediate’ state of inactivation [30]. This conformational state is distinct from the fast inactivation state, requiring a prolonged period of hyperpolarization to recover from. Consequently, the slow inactivation process may influence channel availability at fast heart rates [24]. Investigation of the development of slow inactivation revealed that the fraction of channels (A) that slow inactivated at the end of a 1000 ms depolarization step was significantly reduced for 1493delK sodium channels (A  = 0.22, calculated as 1 minus normalized I_Na_) with respect to WT sodium channels (A  = 0.37) (Figure 4C, Table 3). Besides, the 1493delK mutation was also responsible for a 4.5-fold decrease in the rate of development of slow inactivation (*τ*) (Figure 4C, Table 3).

Summarizing, the kinetic changes observed for the 1493delK mutant sodium channel, i.e. a reduction of both the fast and slow inactivation rate and a hastening of recovery from inactivation, will lead to a gain-of-function.

### Immunocytochemistry of SCN5A-wild-type and SCN5A-1493delK

**Figure 5:**
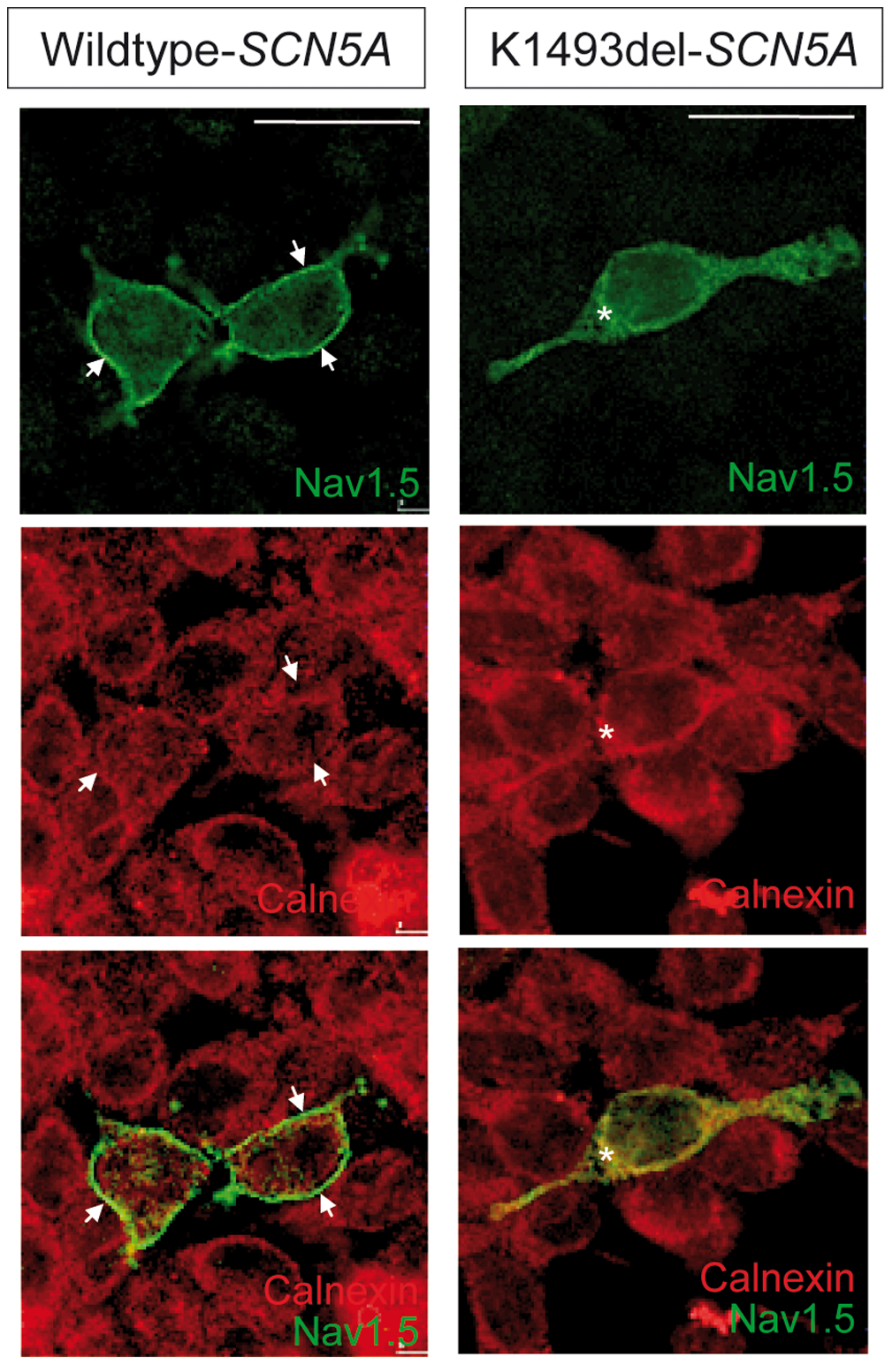
Sodium channel membrane expression in wild-type and mutant 1493delK *SCN5A*-transfected HEK293 cells. Confocal immunofluorescence of the a-subunit of cardiac sodium channel (Na_V_1.5) and the endoplasmic reticulum transmembrane protein calnexin in HEK293 expressing WT (left) and mutant 1493delK (right) sodium channels. Top and middle panels show staining with anti-Na_V_1.5 (green) and anti-calnexin (red) respectively. Bottom panels show overlay of red and green channels of double staining with anti-Na_V_1.5 (green) and anti-calnexin (red) antibodies. Membrane labeling for Na_V_1.5 is observed as a clearly distinguishable green rim surrounding the intracellularly located calnexin (red) in WT *SCN5A* transfected HEK293 cells, whereas mutant 1493delK *SCN5A* transfected HEK293 cells do not show clear cell-surface labeling, but mostly cytoplasmic Na_V_1.5 staining. Scale bars indicate 25 µm.

To establish whether the reduction in I_Na_ peak amplitude reflects a reduction in the number of channels in the plasma membrane or is due to the presence of a population of non-functional channels, immunocytochemical analysis was performed in HEK293 cells expressing with WT and 1493delK sodium channels. Transfected HEK293 cells were stained with antibodies against the cardiac sodium channel protein Na_V_1.5 and the endoplasmic reticulum (ER) transmembrane protein calnexin. Cells transfected with WT *SCN5A* showed a clearly distinguishable rim of Na_V_1.5 staining surrounding the intracellular calnexin staining, indicating successful trafficking of sodium channels to the cell surface (Figure 5). In contrast, cells transfected with 1493delK mutant *SCN5A* showed little discernable membrane labelling, but merely diffuse intracellular staining for Na_V_1.5, which showed a similar pattern as ER protein calnexin. Thus, these results indicate that the 1493delK mutation causes reduced peak sodium current by interfering with normal trafficking of sodium channels to the cell membrane.

## Discussion

Here we report on a large family with a high rate of SCD and a *SCN5A* mutation (p.1493delK) that is located within the linker region between the domains DIII and DIV, which is responsible for the inactivation of the channel [27]. Mutations in *SCN5A* have been associated with various electrical heart diseases including LQTS [9], BrS [10], sinus node dysfunction [15], atrial fibrillation [13], [14], atrial standstill [16] and (progressive) CCD [11], [12], [23]. In general, *SCN5A* mutations leading to a decrease in sodium current (loss-of-function) have been associated with conduction slowing and therefore genetically linked primarily to BrS and (progressive) CCD [6]. Gain-of-function mutations in *SCN5A* are typically associated with LQTS. Although initially the different *SCN5A* mutations were linked to separate disorders, meanwhile numerous reports have shown that a single sodium channel mutation may inflict various combinations of clinical phenotypes of these disorders [31], [32], [33].

### Clinical Phenotype of 1493delK Mutation Carriers

The identified *SCN5A* p.1493delK mutation carriers showed no signs of LQTS or BrS phenotype (even after ajmaline challenge), but clear incidence of CCD consisting of increased P-wave duration, AV-block I° and/or intraventricular conduction slowing in 12-lead rest ECG. Overall, five patients received an ICD for primary prevention and one patient a pacemaker. The pacemaker was implanted at the age of 38 years due to sick sinus syndrome and sinus arrest, 10 years later the patient died suddenly. Since the exact circumstances are unclear, ventricular arrhythmia cannot be ruled out. Also, in one (10021_154) out of the five patients with an implanted ICD, a ventricular arrhythmia occurred, which was terminated by an ICD shock. Overall gross structural changes were not seen in *SCN5A* mutation carriers [28]. Taking together, the identified *SCN5A* p.1493delK mutation leads to CCD, ventricular arrhythmias and SCD, in the absence of signs of BrS or LQTS.

### Biophysical Properties and Membrane Expression of 1493delK Mutant Sodium Channels

To investigate the correlation between the clinical and genetic findings, electrophysiological studies were performed. Patch clamp analyses of HEK293 cells transfected with *SCN5A* 1493delK showed 5-fold reduced peak I_Na_ with altered inactivation kinetics compared to WT transfected cells. Whereas equilibrium properties of inactivation (and activation) were not affected, there was a profound reduction in fast inactivation rate, as well as a reduction in the fraction of channels that fast inactivated. The “intermediate” or “slow” inactivation process was similarly affected. Moreover, the deletion of K1493 produced a hastening of the recovery from inactivation. In theory, all these kinetic changes will lead to an increase of sodium current, and thus cannot account for the significant decrease in 1493delK current amplitude we observed.

Immunostaining experiments further identified that wild type Na_V_1.5 was homogenously expressed within the sarcolemma, whereas 1493delK Na_V_1.5 was primarily located intracellularly, displaying a similar expression pattern as the endoplasmic reticulum (ER) marker calnexin (Figure 5). This presumes a trafficking defect comprising premature degradation of the greater part of the mutant protein by the ER quality control system, leading to reduced expression of the mutant channel in the sarcolemma.

Thus, the 1493delK mutation shows both loss-of-function, i.e. reduced sarcolemmal expression, and gain-of-function aspects, i.e. slowed inactivation kinetics. Since carriers of the 1493delK mutation present with CCD in the absence of a LQTS phenotype, it must be concluded that the mild increase in current due to slowed inactivation is not sufficient to prolong QT-intervals and that the loss-of-function properties dominate [34]. Hence, the profound reduction in 1493delK sodium channel expression results in a lower action potential upstroke velocity with consequent reduction in conduction velocity, as has also been described for other mutations linked to CCD [11], [35], [36].

Although a mutation in the cardiac sodium channel gene is highly compatible with the clinical phenotype of this family, we cannot rule out a contribution of mutations in other genes, e.g. SCN1B [37].

### Comparison with other Sodium Channel Mutations Located in the DIII–DIV Linker

**Figure 6:**
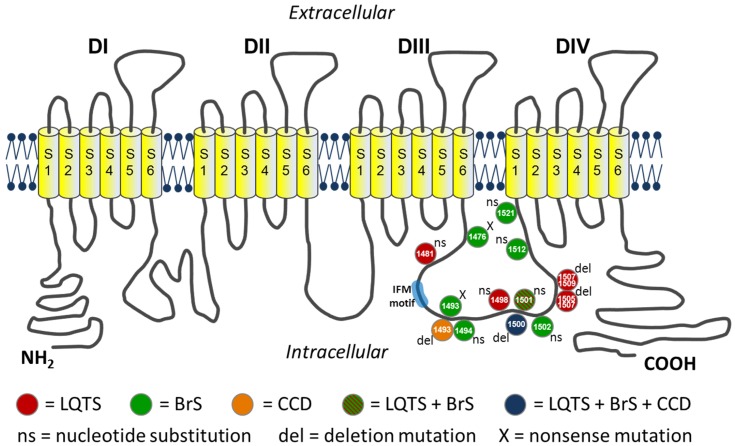
Topological model of the cardiac sodium channel (Na_V_1.5). Location of the mutations in the linker region between domains DIII and DIV that is responsible for the inactivation of the channel.

The 1493delK mutation is located in the loop linking domain III and IV of the sodium channel α-subunit, which mediates fast inactivation by occluding the intracellular side of the channel pore shortly following activation [27], [38]. Critical to fast inactivation is the hydrophobic IFM motif (isoleucine–phenylalanine–methionine), the inactivation particle, formed by residues 1485 through 1487. The positively charged lysine residue K1493 is located 6 residues downstream of this IFM motif and forms part of a cluster of charged residues contained within a α-helical structure. The DIII–DIV linker is rich of clusters of charged residues, which are not essential to inactivation, but may modulate deactivation and fast inactivation kinetics [39]. Over fifteen *SCN5A* mutations have been identified in the DIII–DIV linker (Figure 6), leading to either LQTS (e.g. p.G1481E, p.M1498T, p.1500delK, p.L1501V, p.1505–1507delKPQ, p.1507–1509delQKP) [40], [41], [42], [43] due to gain-of-function as a result of residual current [33] or to BrS (e.g. p.Q1476X, p.K1493X, p.Y1494N, p.1500delK, p.L1501V, p.G1502S, p.R1512W, p.I1521K) [23], [44], [45], [46], [47] as a result of reduced availability and/or slowing of recovery from inactivation [33] leading to loss-of-function. Of note, the 1500delK and L1501V [43], [48] mutations are associated with overlap syndromes of LQTS and BrS (L1501V), and LQTS, BrS and CCD (1500delK) respectively.

In the set of DIII–DIV linker mutations (Figure 6), two contain a deletion (or substitution) of a single positively charged residue, ditto the 1493delK. The 1500delK [48], located in proximity to the 1493delK contained in the same α-helix downstream of the IFM motive, and the R1512W [47], [49]. Comparing the functional consequences of these mutant channels with the 1493delK (this study) reveals both differences and similarities. While sodium current density was unaltered for 1500delK and R1512W, we found a profound reduction in peak 1493delK I_Na_ amplitude. Of note, a double mutation at the analogous position in the rat brain sodium channel SCN3A (KK1441/1442NN), similarly showed reduced expression [50]. Additionally, the shifts in voltage-dependence of activation and steady-state inactivation as reported for 1500delK and R1512W, were absent in 1493delK. However, the slowing of fast inactivation at voltages positive to ∼–40 mV is a common property to all 3 mutations. Enhancement of a late current component was only present in the 1500delK mutant channel, which explains the LQT phenotype in addition to BrS and CCD in carriers of this mutation.

### Conclusion

The deletion mutation 1493delK in the *SCN5A* gene predisposes to conduction disease, ventricular arrhythmias and SCD with a phenotype of P-wave prolongation, AV-block I° and unspecific intraventricular conduction delay, without evidence for Brugada syndrome. The critical underlying mechanism is a five-fold reduction of sodium current due to a trafficking defect of mutant Na_V_1.5 from the ER to the sarcolemma.
